# Albuca Bracteata Polysaccharides Attenuate AOM/DSS Induced Colon Tumorigenesis via Regulating Oxidative Stress, Inflammation and Gut Microbiota in Mice

**DOI:** 10.3389/fphar.2022.833077

**Published:** 2022-02-21

**Authors:** Ziyan Qin, Xinyu Yuan, Jian Liu, Zhuqing Shi, Leipeng Cao, Lexuan Yang, Kai Wu, Yongliang Lou, Haibin Tong, Lei Jiang, Jimei Du

**Affiliations:** ^1^ Department of Microbiology and Immunology, School of Laboratory Medicine, Wenzhou Medical University, Wenzhou Key Laboratory of Sanitary Microbiology, Wenzhou, China; ^2^ College of Life and Environmental Science, Wenzhou University, Wenzhou, China; ^3^ Central Laboratory, School of the First Clinical Medicine and the First Affiliated Hospital of Wenzhou Medical University, Wenzhou, China; ^4^ Laboratory Animal Center, Wenzhou Medical University, Wenzhou, China

**Keywords:** colitis-associated colorectal cancer, CAC, polysaccharides, anti-inflammation, anti-oxidant, anti-tumor, gut microbiota, short-chain fatty acid

## Abstract

Inflammation is an important risk factor in the development of inflammatory bowel disease (IBD) and colitis-associated colorectal cancer (CAC). Accumulating evidence indicates that some phytochemicals have anti-cancer properties. Polysaccharides extracted from Albuca bracteata (AB) have been reported to possess anti-neoplastic activities on colorectal cancer (CRC) models. However, it is still unclear whether they exert therapeutic effects on colorectal cancer. In this study, we investigate the properties of polysaccharides of A. bracteate, named ABP. The average molecular weight of ABP was 18.3 kDa and ABP consisted of glucose, mannose, galactose, xylose, galacturonic acid, glucuronic acid at a molar ratio of 37.8:8:2.5:1.7:1:1. An Azoxymethane/Dextran sodium sulfate (AOM/DSS) induced CAC mouse model was established. The CAC mice treated with ABP showed smaller tumor size and lower tumor incidence than untreated ones. ABP increased anti-inflammatory cytokine IL-10, inhibited secretion of pro-inflammatory cytokines (IL-6, IFN-γ, and TNF-α), mitigated oxidative stress by increasing GSH and decreasing MDA levels, suppressed the activation of STAT3 and expressions of its related genes c-Myc and cyclin D1. Moreover, ABP treatment increased the relative abundance of beneficial bacteria (*f_*Ruminococcaceae, *g_Roseburia, g_Odoribacter, g_Oscillospira,* and *g_Akkermansia*) and the levels of fecal short-chain fatty acid (SCFA) in CAC model mice. In summary, our data suggest that ABP could be a potential therapeutic agent for treating CAC.

## Introduction

Colorectal cancer (CRC) is the third most common cause of tumor-related death worldwide (Siegel et al., 2021). Accumulating evidence reveals that inflammation is a vital factor that contributes to CRC development and growth, and inflammatory bowel disease (IBD) is a known risk factor for colitis-associated colorectal cancer (CAC) (Ullman and Itzkowitz, 2011; Robles et al., 2016). The Azoxymethane/Dextran sodium sulfate (AOM/DSS) mouse model is commonly used for studying colitis-related carcinogenesis and cancer-preventive intervention. Application of the AOM/DSS model has been used to unravel the pathogenesis of CAC from the perspectives of signaling pathways (Fukata et al., 2007; Grivennikov et al., 2009), anti-oxidant machinery (Barrett et al., 2013) and the influence of gut microbiota (Uronis et al., 2009).

CAC development is closely associated with gut flora (Kang and Martin, 2017), and the gut microbiota of CAC is altered with decreased probiotic bacteria (Li et al., 2019) concurrently with enriched pathogenic and opportunistic bacteria (Chattopadhyay et al., 2021). Dysbiosis of gut microbiota increases intestinal permeability, allowing microbial products and microbes to translocate from intestinal lumen to mucosa. After recognizing microbes *via* the Toll-like receptor, intestinal immune cells and epithelial cells activate downstream molecules of inflammatory signaling pathways, including STAT3 (Waldner and Neurath, 2015). The transcriptional activity of STAT3 involves multiple cellular processes, such as cell survival, proliferation, angiogenesis, and immune evasion (Yu et al., 2009; Yu et al., 2014). Accumulation of pathogenic bacteria ultimately provides a proinflammatory environment that favors tumor promotion (Papapietro et al., 2013). While probiotics like *Lactobacillus* or *Bifidobacterium* exerts anti-cancer roles by inhibiting inflammation and angiogenesis, and modulating the intestinal barrier function by generating short-chain fatty acids (SCFAs) (Zhong et al., 2014). Numerous studies have indicated that gut microbiota plays a critical role in host metabolism and immune system development. Thus, regulating the balance of gut microbiota may be therapeutically viable and promising for IBD and CAC treatment (Song et al., 2018).

Polysaccharides have attracted increasing attention for their therapeutic activity in cancer treatment with few side effects. Several polysaccharides have been used to prevent and treat CAC (Sanders et al., 2016; Ji et al., 2018). Studies have shown that polysaccharides attenuate intestinal mucositis with immuno-modulatory, various pharmacological effects and anti-neoplastic effects (Liu et al., 2018b; Ren et al., 2018). Zou et al. reported that *Ficus carica* polysaccharides elevated the expression of tight junction protein Claudin-1 and inhibited the formation of cytokines (TNF-α and IL-1β) to prevent DSS-induced colitis in C57BL/6J mice (Zou et al., 2020). Liu et al. reported that tea polysaccharides inhibited colon tumorigenesis in mice by regulating signaling pathways (Liu et al., 2018c).

*Albuca bracteata* *(Thunb.)* *J.C.Manning* and *Goldblatt* (AB) has long been cultivated in China and used as an herbal remedy for diabetes, hepatitis, cancers, and other diseases (Zhang et al., 2017). In many parts of China, particularly in the southeast region, bulbs of AB are commonly used as a dietary therapy for improving anti-tumor immunity and relieving tumor-induced pain or side effects of chemotherapy. In a previous study (Yuan et al., 2021), ABP showed anti-tumor effects on a homograft CRC mouse model, indicating that ABP may be a potential anti-tumor therapeutic agent for treating CRC. However, it is still unclear whether ABP exert therapeutic effects on CAC.

In this study, the molecular composition of ABP was analyzed, and treatment effects and potential mechanisms of ABP against tumor were explored in an AOM/DSS-induced CAC mice model. The results showed that ABP exhibited various bio-activities, including anti-inflammation, anti-oxidant, regulating gut microbiota, and anti-tumor effects. ABP could be a valuable therapeutic agent for CAC and a good candidate for agents in medicine and functional foods.

## Materials and Methods

### Extraction of ABP

ABP was extracted and purified as previously described by Chen et al. (Chen et al., 2012) with minor modifications. Briefly, bulbs of AB were washed and sliced into small pieces, dried at 60°C for 48 h and then ground in a high-speed disintegrator. The dried AB powder (particle size: 0.6 mm) was soaked overnight in 95% ethanol at a ratio of 1:10 (weight/volume) to remove oligosaccharides, small molecule chemicals and colored materials. Then the pretreated powder was oven-dried at 60°C overnight. The powder was extracted with distilled water for 3 h using the Soxhlet apparatus at 95°C, and the extracts were collected, filtrated, and concentrated using a rotary evaporator. ABP was precipitated by adding ethanol to the final concentration of 75% by volume, and the ABP was collected after centrifugation and dried under nitrogen gas flow.

### Analysis of Chemical Compositions

The total carbohydrate content of ABP was determined by the phenol sulfuric acid method (Liu et al., 2020). The uronic acid content was quantified *via* the *m*-hydroxydiphenyl method as previously described by Liu et al. (2021b) using d-glucuronic acid as the standard. The protein content was measured using Bradford’s method (Wu et al., 2019). The characteristics of chemical groups and bonds in ABP were determined using the Fourier transform-infrared (FT-IR) spectroscopy (BRUKER Tensor 27, Ettlingen, Germany) and KB-disk method and recorded in the frequency range of 4,000–500 cm^−1^.

### Molecular Weight of ABP

The average molecular weight (Mw) of ABP was measured via high-performance gel-permeation chromatography (HPGPC) using an Agilent 1,260 Infinity Ⅱ HPLC system, equipped with a TSK-GEL G4000 PW_XL_ column (Φ7.8 mm × 300 mm, TOSOH, Japan) and the Agilent 1260-RID detector, eluted with Na_2_SO_4_ solution (0.1 M). A standard curve of molecular weight was established using Dextran standards (180 Da, 4.6 kDa, 7.1 kDa, 21.4 kDa, 41.1 kDa, 150 kDa, and 2000 kDa).

### Monosaccharide Composition

The monosaccharide composition of ABP was analyzed based on a previously described method (Wu et al., 2019) with some modifications. Briefly, the sample ABP was hydrolyzed in trifluoroacetic acid (2 M) at 120°C for 3 h. The filtered hydrolysate was analyzed by an Agilent 1,260 Infinity Ⅱ HPLC system equipped with a Hypersil ODS-2 column (5 μm, 4.6 mm × 250 mm) and Agilent 1260-DAD detector. The optimal analysis conditions were set as follows. The eluent was 0.05 M phosphate buffer solution (pH 6.8) and acetonitrile (83:17, v/v), the flow rate was 0.8 ml/min, injection volume was 10 μl, monitored absorbance was 254 nm.

### Animal Studies

Animal experiments were performed according to protocols approved by the Institutional Animal Care and Use Committee of Wenzhou Medical University (wydw 2021–0224). Female BALB/c mice (4–5 weeks old) purchased from Laboratory Animal Center of Wenzhou Medical University were randomly divided into four groups: the normal group, A/D group (AOM/DSS), ABP-L group (AOM/DSS +0.5 mg/ml ABP), and ABP-H group (AOM/DSS +1 mg/ml ABP). On the first day of the experiment, mice in the A/D, ABP-L, and ABP-H groups received an intraperitoneal injection of AOM (10 mg/kg), while those in the normal group received physiological saline. After the administration of sterile water for 1 week, 2% DSS was added into the drinking water of the A/D, ABP-L, and ABP-H groups for another week, followed by sterile drinking water for 2 weeks. This DSS water cycle was repeated thrice. Mice in the normal group received sterile water throughout the study. Once the DSS cycles finished, mice in the ABP-L and ABP-H groups received ABP (0.2 ml/per mice) *via* oral gavage for 11 weeks, while those in the A/D group received an equivalent volume of sterile drinking water. During the experiment, body weight of all mice was recorded. Mice were sacrificed using anesthesia, and colorectal tissue, mucosa, fecal and serum of individual mouse were collected after three or 11 weeks of treatment. Serum was used for ELISA experiments, fecal was used for the analysis of SCFAs, colon mucosa was used for 16S rRNA gene sequencing. A portion of colon tissue fixed in 4% paraformaldehyde was prepared for hematoxylin-eosin (HE) staining, and the other portion was stored at -80°C for subsequent GSH, MDA and Western blot assays.

### Western Blot

Western blots were performed as described before (Luo et al., 2021). The primary antibodies used were as follows: anti-Cyclin D1 (#ab40754, Abcam, Cambridge, MA, United States), anti-c-Myc (#ab32072, Abcam, Cambridge, MA, United States), anti-P-STAT3 (#9145, Cell Signaling Technology, Danvers, MA,United States), anti-STAT3 (#12640, Cell Signaling Technology, Danvers, MA,United States), anti-COX-2 (#AF7003, Affinity Biosciences, Cincinnati, OH, United States), and anti-GAPDH (#5174, Cell Signaling Technology, Danvers, MA,United States).

### 16S rRNA Gene Sequencing and Microbiome Analysis

Samples were collected, and total genomic DNA was extracted as described previously (Zhang et al., 2020; Lou et al., 2021). Bacterial sequencing of 16S rRNA genes was performed with the Illumina HiSeq6000 platform (Hangzhou Guhe Information and Technology Co., Ltd., Zhejiang, China). Mcirobiome analysis was done using Quantitative Insights Into Microbial Ecology (QIIME2, V.2020.6) pipeline, PICRUSt and the Statistical Analysis of Metagenomic Profiles (STAMP) software package V.2.1.3 as described previously (Lou et al., 2021).

### Analysis of Short-Chain Fatty Acid in Feces

The quantitative analysis of fecal SCFAs was determined by a trace ultra gas chromatograph coupled with an ISQ mass spectrometer (TRACE 1310-ISQ, Thermo, MA, United States). Briefly, fecal samples were homogenized with 50 μl of 15% phosphoric acid, 100 mg of glass beads, and 100 μl of isocaproic acid as the internal standard for 10 min using a vortex mixer. After acidification, 400 μl of diethyl ether was added to each sample for SCFAs extraction, and then centrifuged the mix for 10 min at 12,000 rpm (4°C). The levels of SCFAs were quantitatively determined by the GC-MS (gas chromatography–mass spectrometry). The GC-MS analysis was performed by a trace ultra GC equipped with an HP-Innowax MS capillary column (30 m × 0.25 mm × 0.5 µm film thickness, Agilent Technologies). Temperature of injector, ion source quadrupole, the GC-MS interface was 250°C, 230°C, 150°C, and 250°C, respectively. The flow rate of helium carrier gas was kept at 1.0 ml/min. Samples were injected (1 µl) with a split injection (split ratio: 10:1). The initial column temperature was 90°C, increased to 120°C at the rate of 10°C/min, then elevated at a rate of 5°C/min to 150°C, and finally increased to 250°C at a rate of 25°C/min and held for 2 min. The mass spectrometer was used in electron impact (EI) ionization mode (70 eV).

### Determination of Reduced Glutathione (GSH) and Malondialdehyde (MDA)

Reduced glutathione (GSH) and malondialdehyde (MDA) were performed to evaluate oxidative stress. GSH was measured by using the GSH assay kit (A006, Nanjing Jiancheng Bioengineering Institute, Nanjing, China) and MDA by MDA assay kit (A003, Nanjing Jiancheng Bioengineering Institute, Nanjing, China). Assays were carried out according to the manufacturer’s instructions.

### Hematoxylin-Eosin Staining and Histological Analysis

Hematoxylin and eosin (HE) staining was performed for histological observation. Colon tissues were paraffin-embedded, dewaxed, rehydrated, and stained with HE. The staining was performed as described previously (Zhu et al., 2021). The histological score of each mouse was record and values ranged from 0 to 10 according to the extent of colon injury. The scoring criteria were presented in Supplementary Table S2.

### Measurement of Cytokine Levels

Serum cytokines were measured and quantified with the Mouse IL-6 ELISA Kit (Catalogue Number: SU-B20012, Konodee Biotechnology Co., Fujian., China), Mouse IL-10 ELISA Kit (Catalogue Number: SU-B20005, Konodee Biotechnology Co., Fujian., China), Mouse TNF-α ELISA Kit (Catalogue Number: SU-B20220, Konodee Biotechnology Co., Fujian., China) and Mouse IFN-γ ELISA Kit (Catalogue Number: SU-B20652, Konodee Biotechnology Co., Fujian., China), according to the manufactures instructions.

### Statistical Analysis

Data were presented as means ± standard deviation (SD) or median. Normally distributed data were analyzed by student’s *t*-test, non-normal distributed data were analyzed using the Kruskal–Wallis test (SPSS 23.0, SPSS Company, Inc., United States). The threshold for significance was *p* < 0.05.

## Results

### Chemical Composition of ABP

**FIGURE 1:**
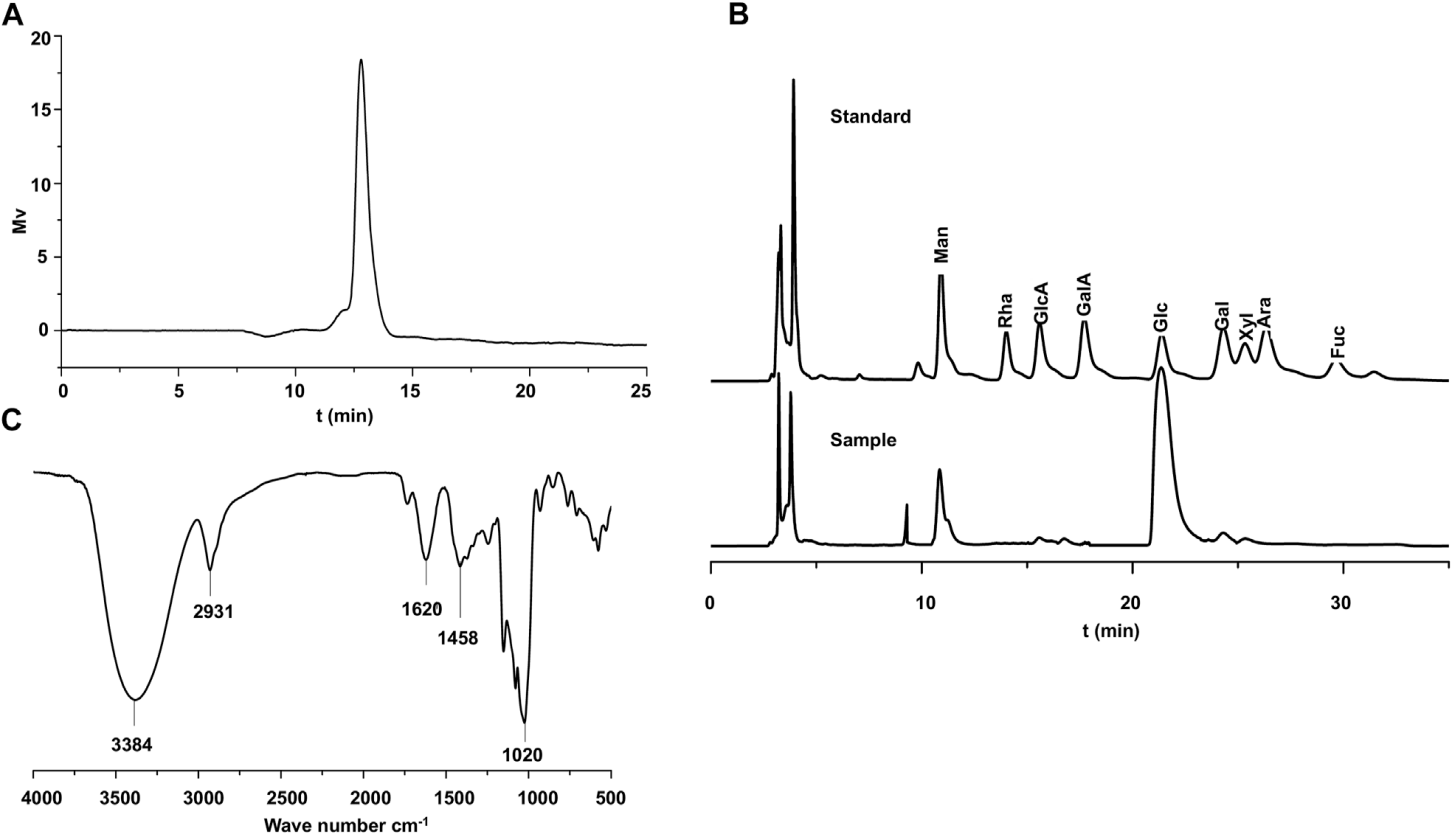
Chemical Composition of ABP. **(A)** HP-GPC profiling of ABP.**(B)** Liquid chromatogram of standard monosaccharide derivatives and liquid chromatogram of hydrolyzed monosaccharide derivatives of ABP. **(C)** FT-IR spectrum of ABP.

The total carbohydrate, uronic acid, and protein contents of ABP were 92.58%, 1.63%, and 1.70%, respectively (**Tabel S1**). Dextran standards with the molecular weight from 180 Da to 2,000 kDa were applied onto an HPLC system to calibrate the column. According to the standard curve Log(Mw) = 2.35–0.421 t_R_ (Mw is the average molecular weight, t_R_ is the retention time, correlation coefficient *R* ^2^ = 0.997), the Mw of ABP was 18.3 kDa, and the HPGPC profile of ABP (Figure 1A) showed a single and symmetrically sharp peak, indicating that ABP was a homogeneous polysaccharide. The composition of component monosaccharides is an important parameter for assessing polysaccharide characteristics. According to the HPLC analysis with a PMP (1-phenyl-3-methyl-5-pyrazolone) pre-column derivatization method, the monosaccharide compositions of ABP were shown in Figure 1B and Supplementary Table S1. Overall, the data indicated that ABP was a hetero-polysaccharide composed of glucose, mannose, galactose, xylose, galacturonic acid, glucuronic acid in a ratio of 37.8: 8: 2.5: 1.7: 1: 1. Glucose is the major monosaccharide. The FT-IR spectrum of ABP (Figure 1C) showed intense absorption peaks at 3,384 cm^−1^ for O-H stretching vibration (Li et al., 2017; Su and Li, 2020) and 2,931 cm^−1^ for C-H stretching vibration (Wang et al., 2012; Hashemifesharaki et al., 2020; Ma et al., 2020). The absorption peak at 1,620 cm^−1^ and 1,458 cm^−1^ were the characteristic absorption peaks of C=O (Cao et al., 2018; Zeng et al., 2020) and C-H groups (Sun et al., 2013; Meng et al., 2017). Furthermore, the peak at 1,020 cm^−1^ corresponded to the stretching vibration of C-O groups (Biao et al., 2020; Hu et al., 2020).

### ABP Treatment Ameliorated AOM/DSS Induced Weight Loss and Colonic Damage

**FIGURE 2:**
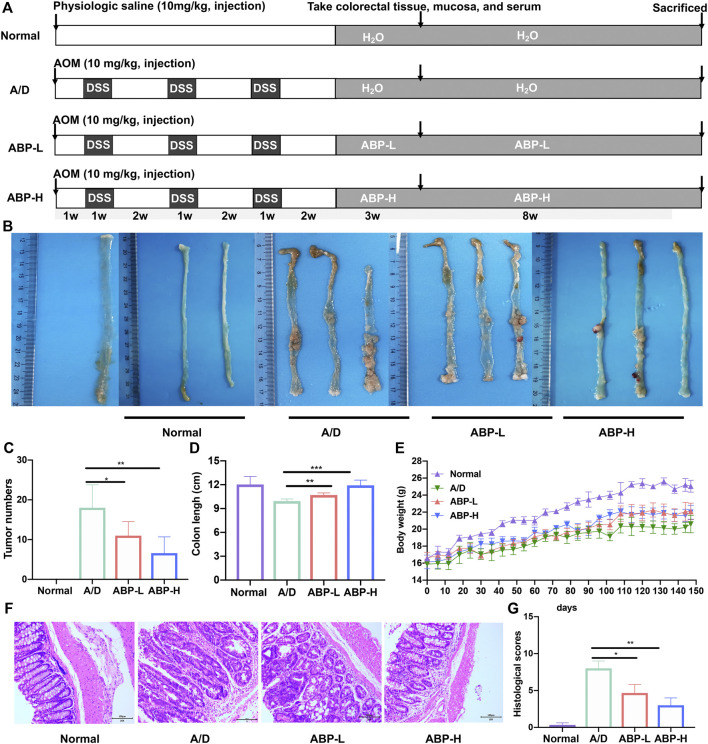
ABP treatment ameliorated weight loss and AOM/DSS-induced pathological damage of colon. **(A)** The overview of the animal experiment design. **(B-C)** Colon tumor burden in each group. **(D)** Colon lengths of mice in each group. **(E)** Body weights of mice in each group. **(F)** Micrographs of HE-stained colon tissues in11 weeks treatment group. **(G)** Histological scores of each group. Compared with the A/D group, *p < 0.05, **p < 0.01.

An AOM/DSS-induced CAC model was established to determine the anti-tumor effect of ABP on CAC mice (Figure 2A). At the end of ABP treatment, compared with the A/D group (18.0 ± 5.788), the ABP-L group (11 ± 3.536, *p* < 0.05) and ABP-H group (6.6 ± 4.099, *p* < 0.01) demonstrated a significant reduction in the number of tumors (Figures 2B,C). Treatment with ABP also alleviated the shortening of the colon (Figure 2D, ABP-L, *p* < 0.01, ABP-H, *p* < 0.001) and weight loss (Figure 2E). Colon tissue sections in the A/D group showed noticeable pathological changes, including colonic epithelial cell destruction, loss of goblet cells, and varying degrees of inflammatory cell infiltration, whereas those of ABP treatment groups displayed a remarkable reduction in symptoms (Figures 2F,G, Supplementary Table S2). These results indicated that intervention with ABP ameliorated weight loss, clinical signs of inflammation and tumor development in AOM/DSS-induced CAC mice.

To evaluate the effect of long-term oral consumption of ABP on the general health of mice, we introduced a group of ABP alone. Results indicated that long-term consumption of ABP did not lead to shortening of the colon (Supplementary Figure S1A, B), loss of body weight (Supplementary Figure S1C), and pathological changes in the colon (Supplementary Figure S1D). Besides, no side effects were observed in the long-term use of ABP.

### ABP Treatment Attenuated the Production of Oxide- and Inflammatory- Cytokines in AOM/DSS-Induced CAC Mice

**FIGURE 3:**
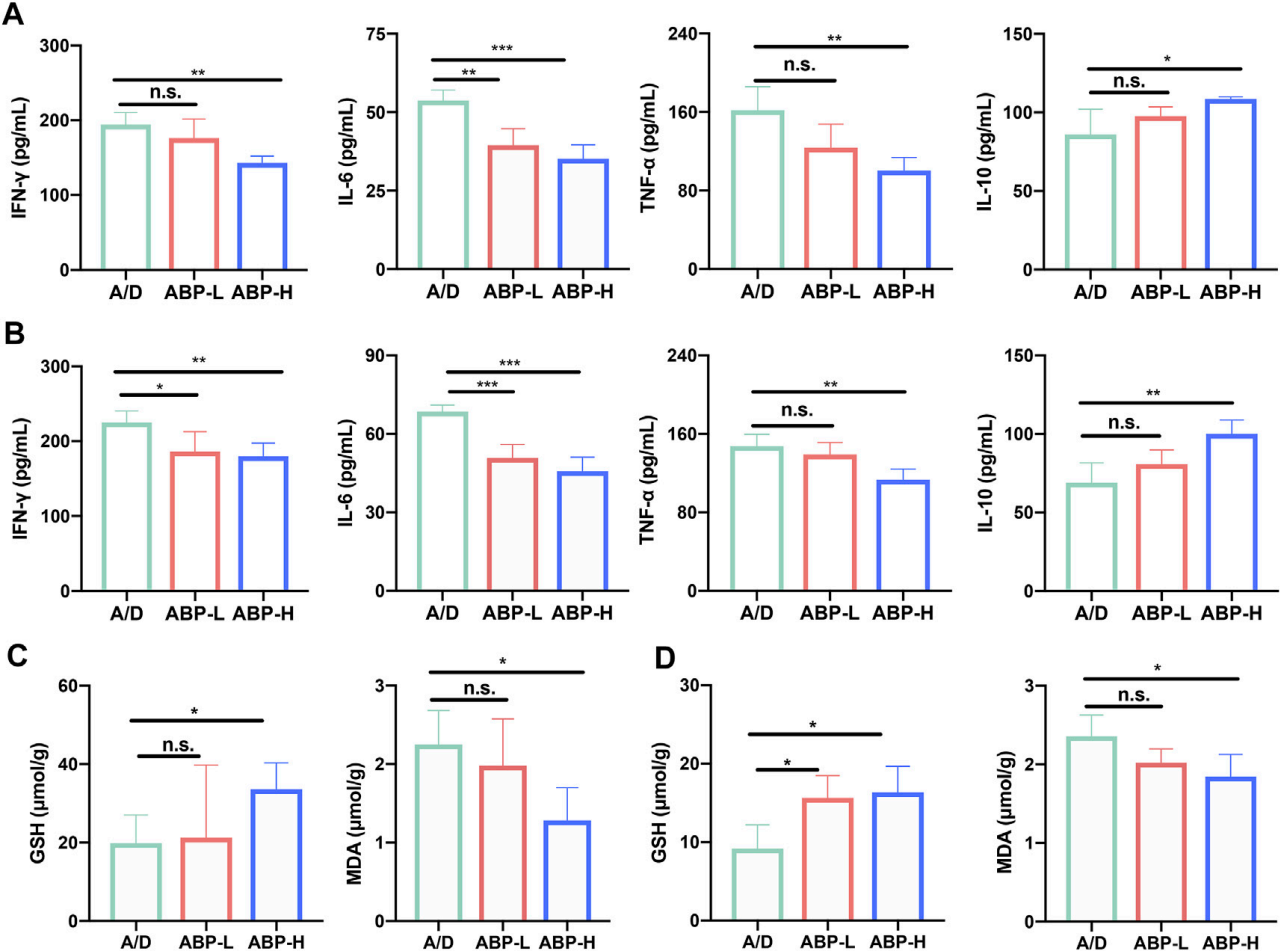
ABP treatment attenuated the production of oxide**-** and inflammatory**-**cytokines of AOM/DSS-induced CAC mice. **(A-B)** Leves of IL-6, TNF-α, IFN-γ, IL-10 in serum of mice treated with ABP for 3 weeks **(A)** and 11 weeks **(B)**. **(C-D)** Levels of MDA and GSH in colon tissue treated with ABP for 3 weeks **(C)** and 11 weeks **(D)**. Compared with the A/D group, *p < 0.05, **p < 0.01.

Because inflammation and oxidative stress might lead to cancer progression, serum levels of inflammatory cytokines and oxidative stress indicators of the colon in CAC mice treated with ABP for three or 11 weeks were detected by ELISA assays and commercial kits. At 3 weeks, the level of pro-inflammatory cytokines TNF-α, IFN-γ, and IL-6 decreased while the anti-inflammatory cytokine IL-10 increased in ABP treatment groups (Figure 3A). A similar phenomenon was also observed at 11 weeks (Figure 3B). An increased level of GSH and decreased level of MDA in the colon were observed in the mice of ABP treatment groups both in 3 weeks (Figure 3C) and 11 weeks of treatment (Figure 3D). These data implied that ABP might in favor of reducing the occurrence and development of CAC by suppressing pro-inflammatory-related cytokines, upregulating anti-inflammatory cytokines, and reducing oxidative damages.

### ABP Treatment Suppressed the Activation of STAT3 in the Colonic Tissue of AOM/DSS-Induced CAC Mice

**FIGURE 4:**
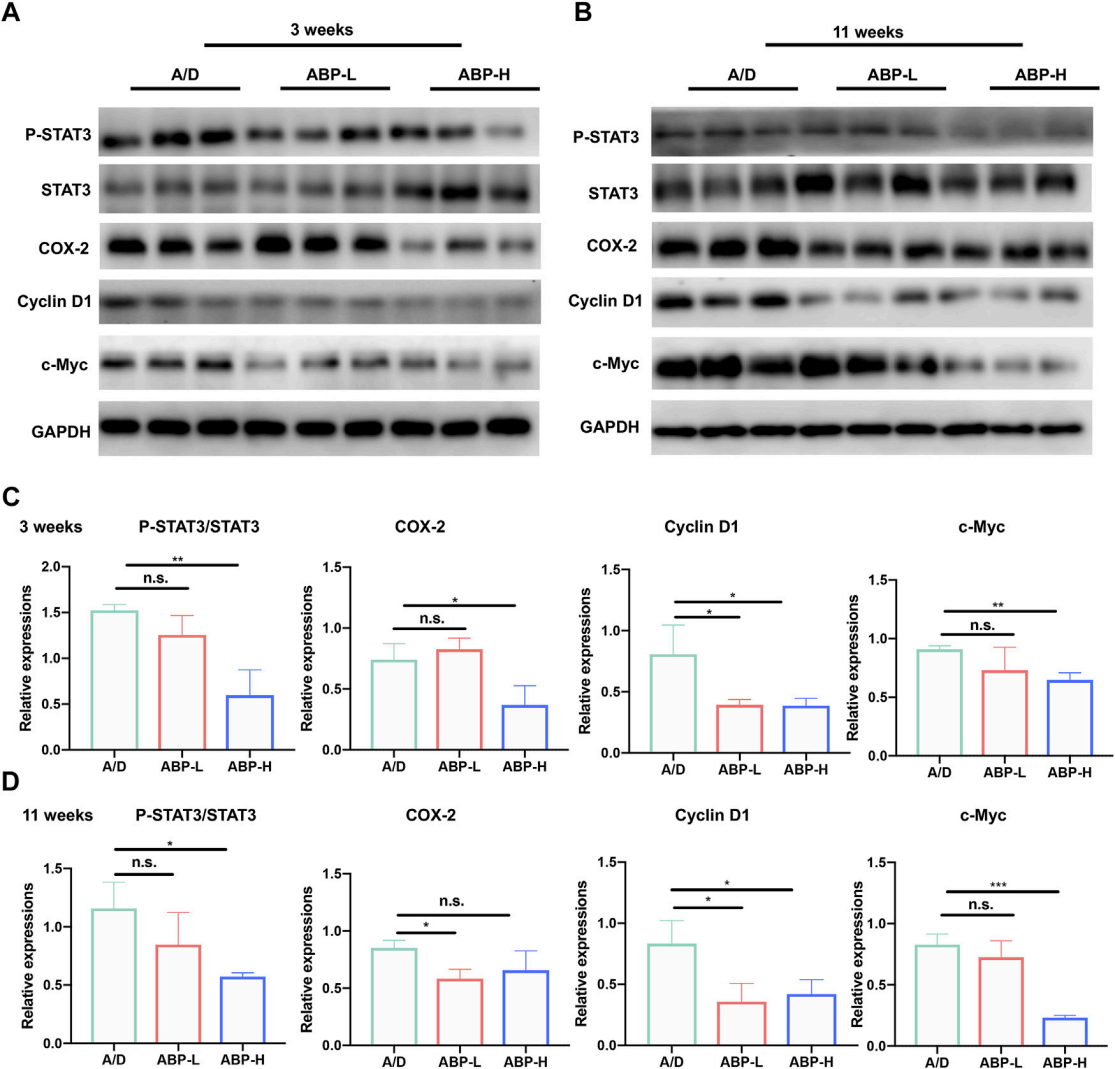
ABP treatment suppressed the activation of STAT3 in the colonic tissue in AOM/DSS-induced CAC mice. **(A-B)** Western blotting of STAT3, P-STAT3, COX-2, Cyclin D1, c-Myc and GAPDH in colon tissue treated with ABP for 3 weeks **(A)** and 11 weeks **(B)**. **(C-D)** P-STAT3 protein expression relative to STAT3. COX-2, c-Myc and Cyclin D1 protein expressions relative to GAPDH in colon tissues treated with ABP for 3 weeks **(C)** and 11 weeks **(D)**. Compared with the A/D group, *p < 0.05, **p < 0.01.

To gain more insights into the regulatory role of ABP in anti-inflammation, we examined proteins related to the IL-6/STAT3 signaling pathway (Figures 4A,B) by Western Blot. It was found that, both in 3 and 11 consecutive weeks of treatment (Figures 4C,D), expressions of P-STAT3, Cyclin D1 and c-Myc were inhibited in ABP treated groups and COX-2 in ABP-H groups. The expression of COX-2 decreased in the 11 consecutive weeks of ABP-L treatment, while no similar trend was found in the 3-weeks group. These results indicated that ABP treatment might regulate expressions of P-STAT3, c-Myc, and Cyclin D1 in CAC mice, suppress the activation of the IL-6/STAT3 signaling pathway and reduce the inflammatory response.

### ABP Treatment Regulated the Intestinal Microflora of AOM/DSS Induced CAC Mice

To reveal ABP treatment effects on gut microbiota composition of AOM/DSS induced CAC mice, 16S rRNA gene sequencing was performed on colon mucosal samples. The gut microbiota diversity and richness were evaluated by Chao1, Shannon, and Simpson indexes.

**FIGURE 5:**
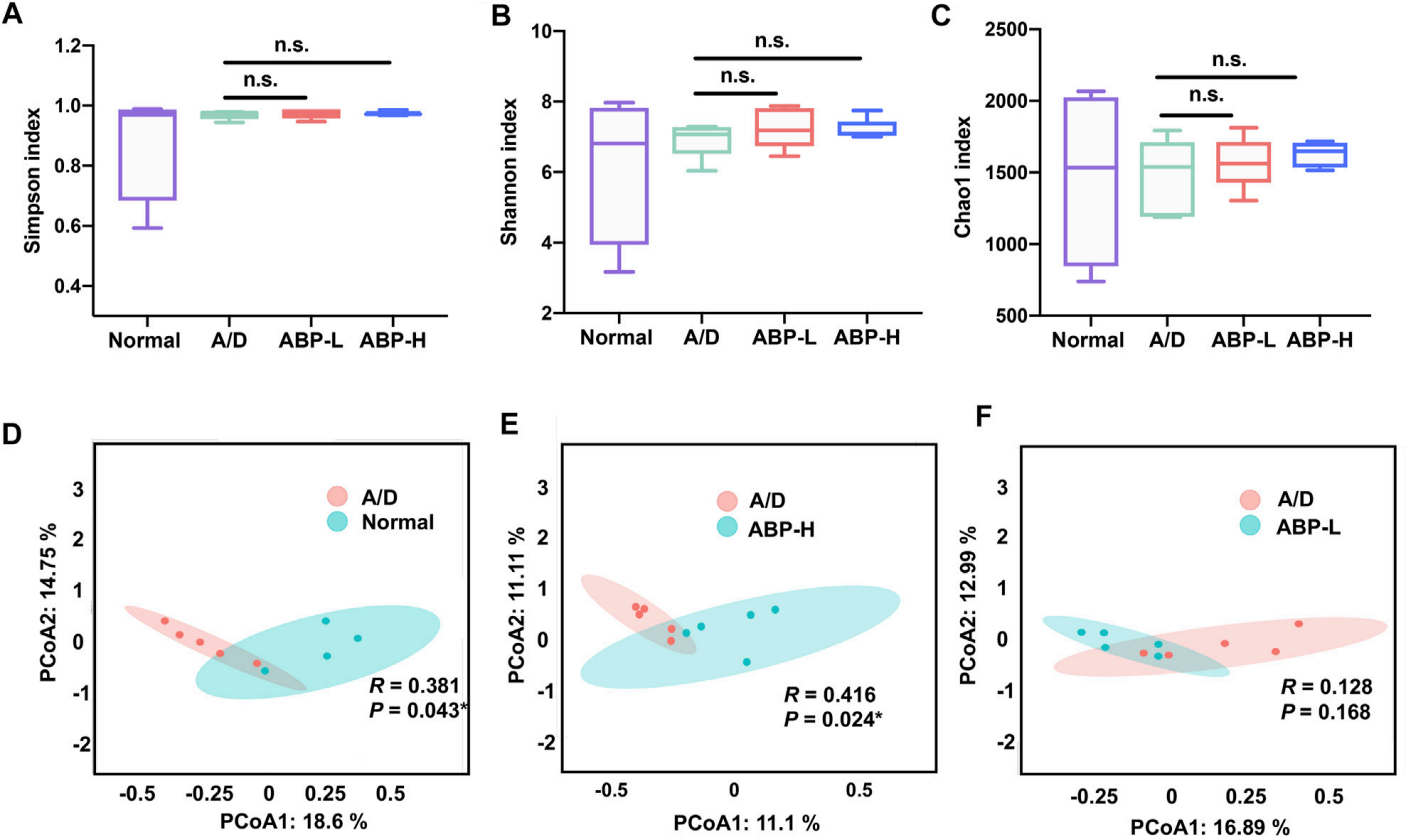
ABP treatment altered the abundance and diversity of gut microbes in AOM/DSS-induced CAC mice (treatment with ABP for 11 weeks). **(A-C)** Analysis of alpha diversity; **(A)** Simpson index. **(B)** Shannon index. **(C)** Chao1 index. **(D-F)** Analysis of beta diversity. principal coordinates analysis (PCoA).

No significant change was found among all groups’ alpha diversity, no matter treated for 3 weeks or 11 weeks (Figures 5A–C, Supplementary Figures S2A, B). The principal coordinates analysis (PCoA) showed an obscure difference between the A/D and ABP groups treated for 3 weeks (Supplementary Figures S2C, D). Compared to the A/D group, the PCoA results showed an apparent difference in the normal group (Figure 5D, *p* = 0.043) and the ABP-H group (Figure 5E, *p* = 0.024) treated for 11 weeks, showed no difference between the ABP-L group and the A/D group (Figure 5F). These results indicated that ABP might influence the intestinal flora composition, and adequate treatment of ABP was required to improve microbiota in CAC mice.

**FIGURE 6:**
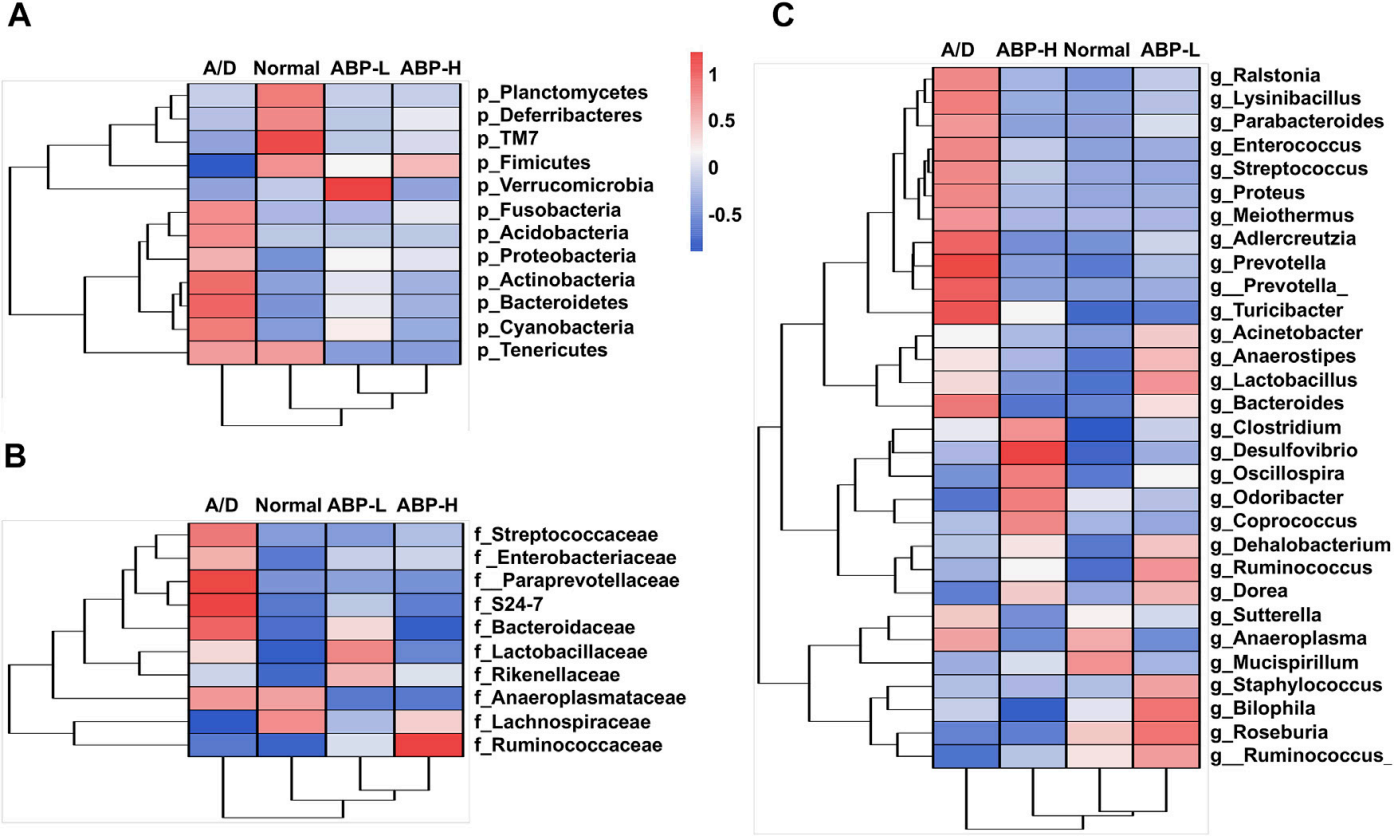
ABP treatment improved the composition of the intestinal microflora in AOM/DSS-induced CAC mice (treatment with ABP for 11 weeks). **(A-C)** Heatmap of bacterial taxa based at the phylum, family and genus levels.

To clarify the effects of ABP on the microflora of CAC model mice, the community composition of each group was analyzed at the phylum, family, and genus levels. The heatmap showed that (Supplementary Figures S3A–C) *g_Lysinibacillus, g_Streptococcus, g_Acinetobacter, g_Gordonia, g_Ochrobactrum, g_Halomonas, and g_Ralstonia* were enriched in the A/D group, while *f_*Rikenellaceae and *g_Turicibacter* were enriched in the ABP-L group treated for 3weeks, *g_Ruminococcus_, g_Enterococcus, g_Odoribacter* and *g_Dehalobacterium* were enriched in the ABP-H group. In the 11- week treatment groups, the heatmap showed that (Figures 6A–C) some potential pathogenic bacteria (*g_Ralstonia*, *g_Proteus*, *g_Adlercreutzia,* and *g_Streptococcus*) enriched in the A/D group*,* while some beneficial bacteria enriched in the ABP-L group (*p_Verrucomicrobia*, *g_Ruminococcus_, g_Roseburia*) and the ABP-H group (*f_*Ruminococcaceae, *g_Oscillospira*, *g_Odoribacte*r, *g_Coprococcus*).

**FIGURE 7:**
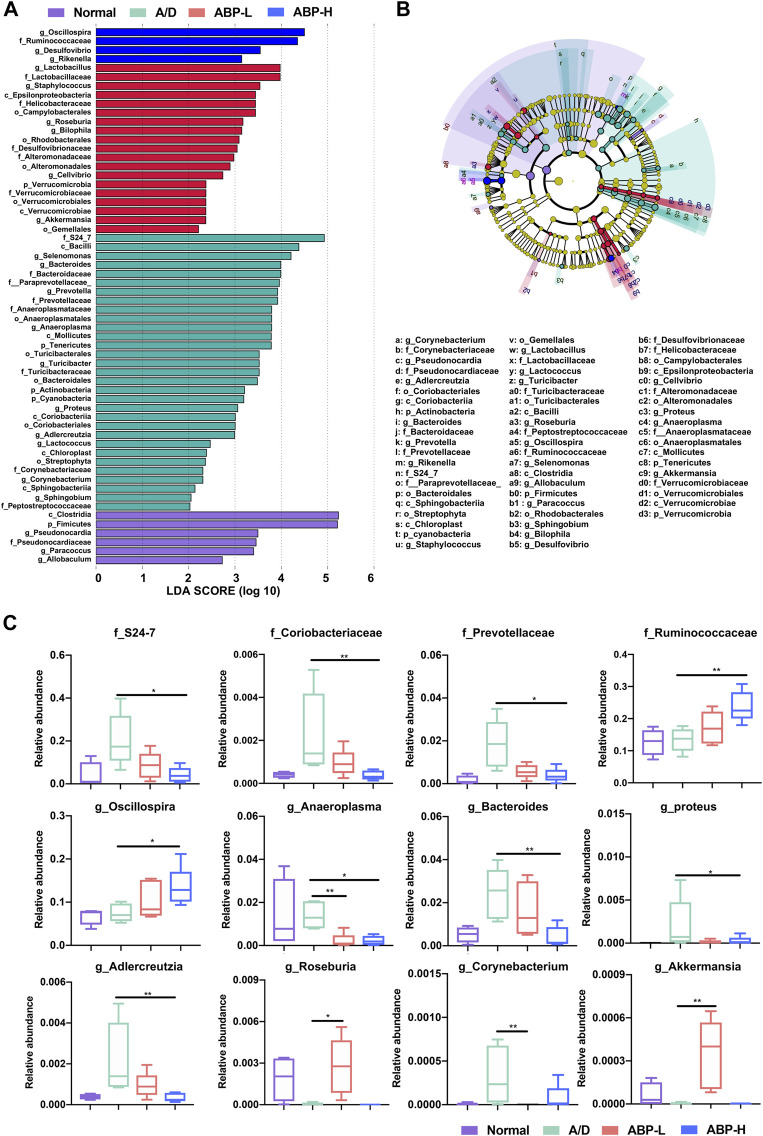
Distribution of colon microbiota in each group (treatment with ABP for 11 weeks). **(A-B)** LEfSe analysis of bacterial taxa differences in each group. **(C)** Box plots demonstrating the characteristic bacteria at the family and genus levels. Compared with the A/D group, *p < 0.05, **p < 0.01.

Differences among groups at family and genus level were also analyzed by liner discriminate analysis (LDA), effect size measurements (LEfSe) and the Kruskal Wallis test (Figures 7A,B, Supplementary Figures S4A, B). The ABP-L group treated for 3 weeks (Supplementary Figure S4C) had higher relative abundance of some bacteria (*g_Turicibacter, p* < 0.01; *g_AF12, p* < 0.01) and lower opportunistic pathogens (*g_Streptococcus, p* < 0.001; *g_Ralstonia, p* < 0.01; *g_Corynebacterium*, *p* < 0.05). The ABP-H group had a higher relative abundance of *g_Odoribacter* (*p* < 0.01) *and g_AF12* (*p* < 0.05), and a less relative abundance of *g_Streptococcus* (*p* < 0.05)*.* The ABP-L group (Figure 7C) treated for 11 weeks had enriched probiotics (*g_Roseburia, p* < 0.05*, g_Akkermansia, p* < 0.01)*,* decreased potential pathogenic and inflammation-promoting bacteria (*g_Corynebacterium*, *p* < 0.01; *g_Anaeroplasma, p* < 0.01)*.* The ABP-H group had much higher relative abundance of *f_*Ruminococcaceae (*p* < 0.01) and *g_Oscillospira* (*p* < 0.05)*,* but less abundance of CAC related bacteria that were enriched in the A/D group (*f_S24-7*, *p* < 0.05; *f_*Coriobacteriaceae*, p* < 0.01; *f_*Prevotellaceae, *p* < 0.05; *g_Bacteroides*, *p* < 0.01; *g_Adlercreutzia, p* < 0.01; *g_Proteus, p* < 0.05 and *g_Anaeroplasma, p* < 0.05).

We also focused on changes of certain bacteria over time. As shown in Supplementary Figure S5, along with the extension of time, the relative abundance of *f_S24-7*, *g_Streptococcus*, *g_Acinetobacter, g_Anaeroplasma* increased in the A/D group. ABP treatment decreased the relative abundance of the four bacteria, increased the relative abundance of *g_Odoribacter, f_*Ruminococcaceae*, g_Oscillospira, g_Coprococcus* gradually. These data proved that ABP might ameliorate gut microbiota dysbiosis, elevate beneficial bacteria levels, and downregulate certain pathogenic bacteria levels in AOM/DSS-induced CAC mice.

### ABP Treatment Improved Fecal SCFA Levels of AOM/DSS Induced CAC Mice

**FIGURE 8:**
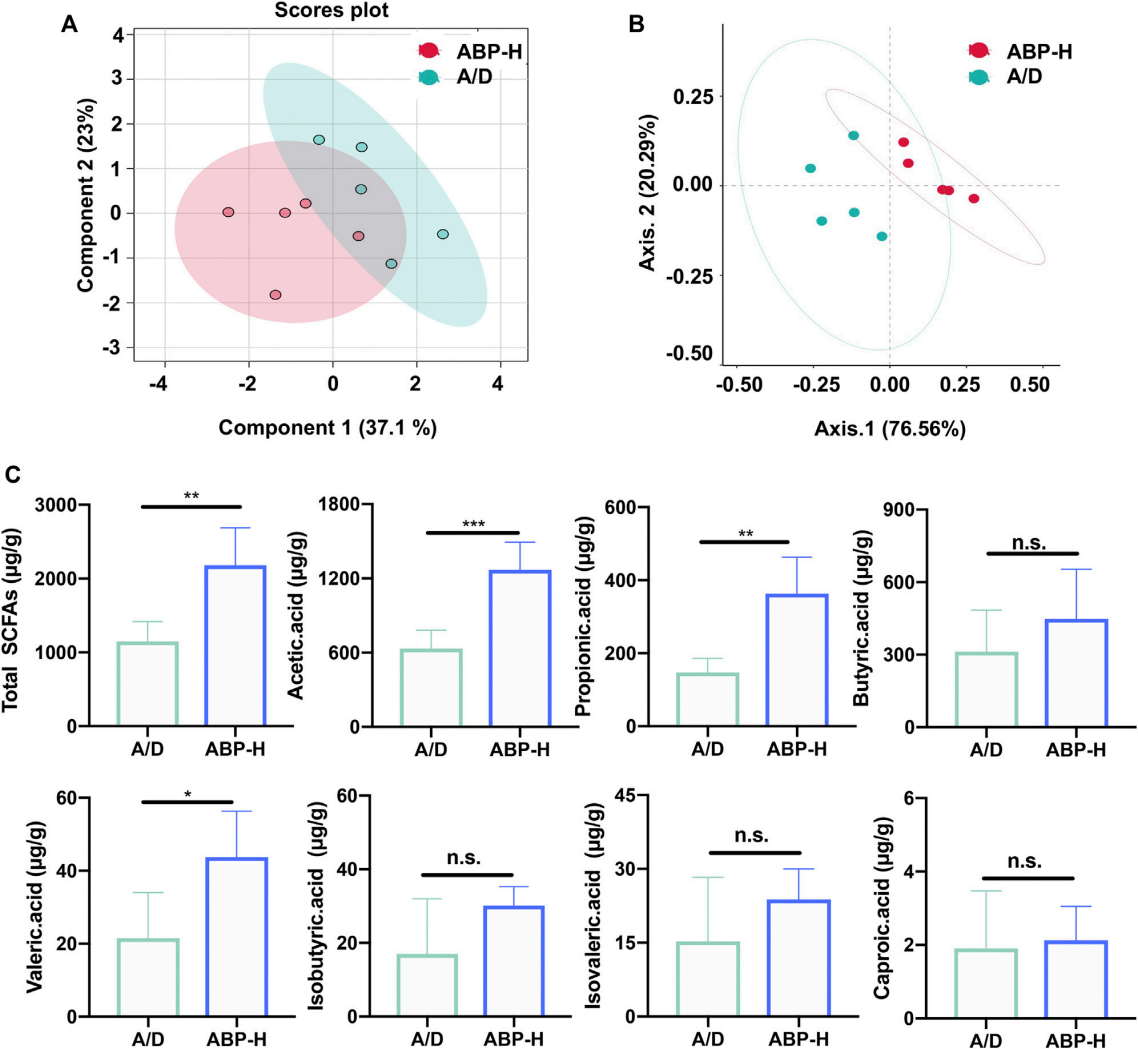
ABP treatment increased the fecal SCFA levels in AOM/DSS-induced CRC mice (treatment with ABP for 11 weeks). **(A-B)** PCoA and PLS-DA analysis. **(C)** Bar plots for fecal SCFA content. Compared with the A/D group, *p < 0.05, **p < 0.01.

The effect of ABP treatment on the metabolism of the intestinal tract was assessed by fecal SCFAs measurement and analysis. PLS-DA and PCoA analysis showed a clear difference between the ABP-H and A/D groups (Figures 8A,B). As shown in Figure 8C, mice in the ABP-H group had a higher concentration of total SCFAs (*p* < 0.01) and individual acetic acid (*p* < 0.001), propionic acid (*p* < 0.01), and valeric acid (*p* < 0.05) than those of the A/D group. These data indicated that ABP could significantly increase the production of SCFAs in mice. The changes in fecal SCFA levels of mice treated with ABP were consistent with the altered composition of the intestinal microbiome.

## Discussion

In mice, colitis induced by DSS is characterized by colon mucosal inflammation accompanied by shortening of the colorectum and body weight loss (Matsunaga et al., 2021). Current research has focused on using natural substances with low toxicity and few side effects on normal cells or organs to offer alternative anti-inflammation and anti-tumor therapies (Giner et al., 2016). Some polysaccharides, such as *Hericium Erinaceus* polysaccharide and *Astragalus* polysaccharide, have been reported to alleviate ulcerative colitis development by suppressing inflammatory cytokines and regulating gut microbiota in animals (Zhao et al., 2016; Ren et al., 2018).

Phytochemical analysis revealed that AB had various components with bio-active properties, such as saponins, flavonoids, polysaccharides, etc. that exhibit anti-oxidant, anti-inflammatory, anti-diabetic, and anti-neoplastic effects (Zhou et al., 2005; Iguchi et al., 2017). In this study, AB was farmed by ourselves and ABP was extracted from bulbs of AB. At first, we determined the Mw, monosaccharide composition and FT-IR spectrum of ABP and the results showed that ABP was a homogeneous polysaccharide with an Mw of 18.3 kDa and mainly composed of glucose, mannose, galactose, and xylose. Next, we confirmed that ABP had antioxidant and anti-inflammatory properties *in vivo*, and ABP treatment could effectively inhibit tumorigenesis and progression by attenuating colon shortening, body weight loss, and histological damages in AOM/DSS induced CAC mice.

Furthermore, we explored the probable mechanisms of ABP against CAC. TNF-α and IL-6 are pro-inflammatory cytokines that play important roles in the process of inflammation (Francescone et al., 2015). The production of these cytokines is regulated by signaling pathways such as the STAT3 pathway. The STAT3 transcription factor induces the expression of cell proliferation related genes (cyclin D1, PCNA) and suppresses apoptotic genes (Bcl-2, Bcl-XL) (Becker et al., 2005; Klampfer, 2008). It is well accepted that STAT3 signaling drives pathological processes, including cell proliferation, recruitment of inflammatory mediators, and angiogenesis (Francescone et al., 2015). Indeed, many inflammatory mediators are positively associated with the prevalence of colorectal adenomas (Kim et al., 2008; Basavaraju et al., 2015; Song et al., 2016). For example, serum levels of IL-6 are higher in CRC patients than in healthy controls (Knupfer and Preiss, 2010). Grivennikov et al. reported that IL-6 promoted colon tumor growth in an AOM/DSS-induced CAC mouse model (Grivennikov et al., 2009). IL-10 is an anti-inflammatory cytokine critical for maintaining intestinal immune homeostasis. IL-10-deficient mice develop intestinal inflammation in the presence of normal gut microflora (Burrello et al., 2018). In this experiment, ABP treatment inhibited the phosphorylation of STAT3, reduced the expression of IL-6 and increased the expression of IL-10, indicating that the anti-inflammatory effect of ABP may be one of its anti-tumor effects.

Oxidative stress has been proposed as a mechanism of IBD. With the production of MDA, excessive NO produces oxygen free radicals that cause tissue damage and the formation of colitis (Zhu and Li, 2012; Li et al., 2016). We found that in CAC mice treated with ABP, MDA contents were significantly decreased and the level of GSH was increased, suggesting that ABP had anti-oxidative capacity and can scavenge free radicals.

The intestinal microbiota plays a pivotal role in physiological homeostasis and pathophysiology of diseases, destruction of intestinal barrier and signals of epithelial cells are closely related to microbiota dysbiosis (Arthur and Jobin, 2013; Jackson and Theiss, 2020). The impaired gut microbiota may result in the damage of enteric mucosa and various inflammation. Therefore, invasive bacteria might cross the barrier and trigger a pro-inflammatory response (Abreu, 2010). As the most commonly used specimen for gut microbiota sequencing, feces may not reflect the real flora of the intestinal surface. There are two main reasons: food consumption and certain bacteria that have penetrated the intestinal barrier cannot be detected precisely, such as *Akkermansia. Akkermansia* plays a critical role in maintaining human immunity and metabolism, can strengthen the intestinal barrier by promoting mucus secretion (van der Lugt et al., 2019), thus it has been deemed a promising therapeutic probiotic (Zou and Chen, 2020; Yu et al., 2021). Hence, we selected mucosal specimens instead of feces in this study.

SCFAs are the main metabolites produced by anaerobic bacteria, which confer healthy outcomes to the host (den Besten et al., 2013; Tan et al., 2014). The beneficial roles of SCFAs for the host include their capacity to strengthen intestinal barrier function, reduce oxidative stress, and anti-inflammatory, anti-carcinogenic effects (Sun and O'Riordan, 2013; van der Beek et al., 2017). Liu et al. reported that pumpkin polysaccharides increased the relative abundance of *g_Oscillospira* altered and increased SCFAs production in diabetic rats (Liu G. et al., 2018). Liu et al. found dietary supplementation of berry anthocyanin extracts enriched levels of SCFA producing bacteria and elevated production of fecal SCFAs in high-fat-fed C57BL/6J mice (Liu et al., 2021a). Xia et al. found that Adaptogenic flower buds increased the relative abundance of SCFA producing bacteria and strengthened the epithelial tight junction complex and immune responses (Xia W. et al., 2020). In this study, we found that ABP treatment enriched the relative abundance of SCFA producing bacteria and elevated fecal SCFA levels in AOM-DSS induced CAC mice.

The heat map and LEfSe analysis showed that ABP decreased the relative abundance of some potentially pathogenic bacteria (*g_Streptococcus, g_Proteus, g_Corynebacterium, g_Anaeroplasma, g_Acinetobacter,* and *g_Ralstonia*) (Wu et al., 2018; Liu et al., 2019; Xia X. et al., 2020; Zorron Cheng Tao Pu et al., 2020; Marongiu et al., 2021; Zhang et al., 2021). GC-MS results revealed that ABP increased the levels of SCFAs in AOM/DSS induced CAC mice. In this study, changes in SCFA levels in ABP treated mice coincided with intestinal microbiome changes.

As expected, the A/D group showed a gradual increase of some bacteria along with time extension, implying these bacteria may be positively related to progression of AOM/DSS induced CAC. In ABP treatment groups, both decreasing trends of relative abundance of CAC related bacteria and increasing trends of SCFA producing bacteria were more apparent in mice treated for 11 weeks than those in 3 weeks, implying that regulation of ABP on the intestinal flora is a continuous and accumulating process. Combined with the long-term use of ABP having no side effect on the ABP alone group, we supposed that ABP could be used for long-term treatment against CAC.

**FIGURE 9:**
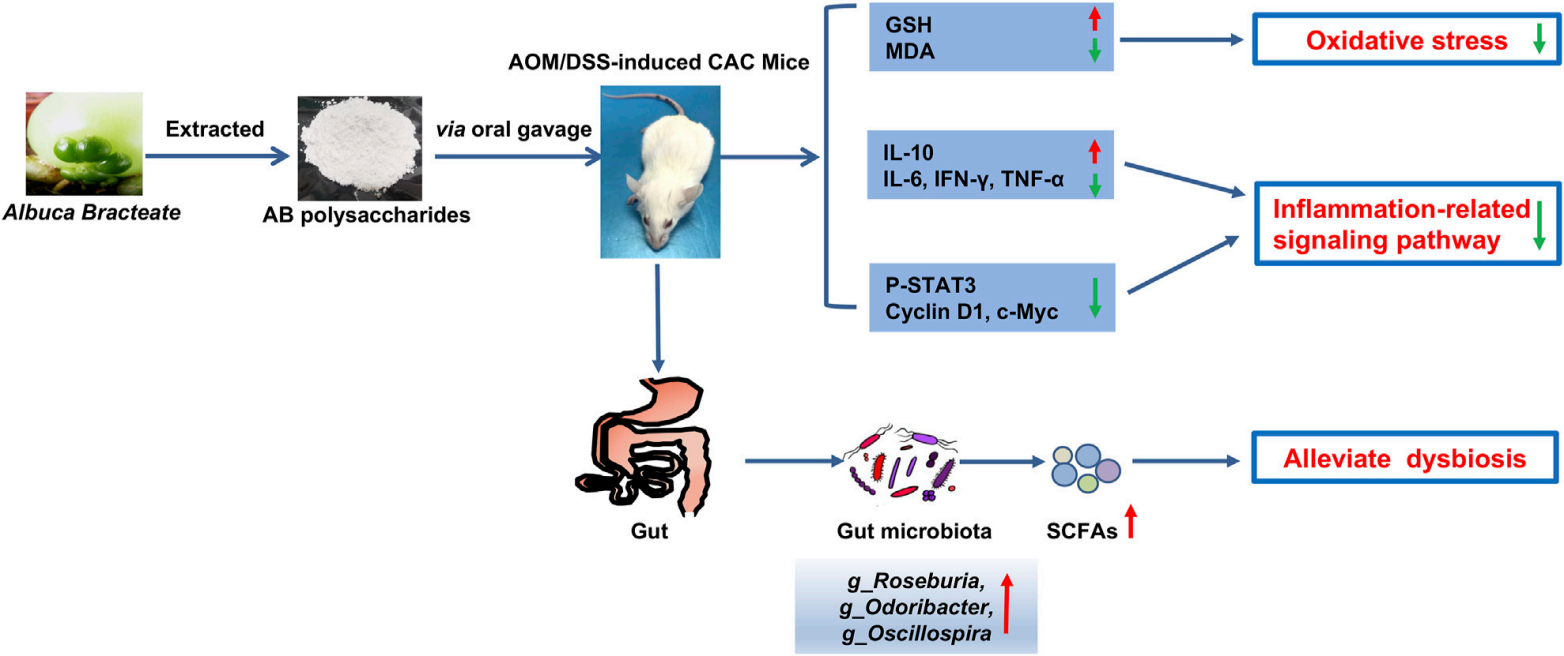
Schematic diagram depicting the mechanism of action underlying the effect of ABP as a regulator of CRC progression.

Taken together, ABP treatment inhibited tumor progression in the AOM/DSS induced CAC mice by improving microbiota, increasing the abundance of beneficial bacteria and fecal SCFAs, reducing oxidative damage in the colon, suppressing inflammatory signaling pathways (Figure 9). ABP may be a potential therapeutic agent for treating CAC.

## Data Availability

The datasets presented in this study can be found in online repositories. The names of the repository/repositories and accession number(s) can be found below: https://www.ncbi.nlm.nih.gov/sra; PRJNA785339.

## References

[B1] Abreu M. T. (2010). Toll-like Receptor Signalling in the Intestinal Epithelium: How Bacterial Recognition Shapes Intestinal Function. Nat. Rev. Immunol. 10 (2), 131–144. 10.1038/nri2707 20098461

[B2] Arthur J. C. Jobin C. (2013). The Complex Interplay between Inflammation, the Microbiota and Colorectal Cancer. Gut Microbes 4 (3), 253–258. 10.4161/gmic.24220 23549517 PMC3669172

[B3] Barrett C. W. Ning W. Chen X. Smith J. J. Washington M. K. Hill K. E. (2013). Tumor Suppressor Function of the Plasma Glutathione Peroxidase Gpx3 in Colitis-Associated Carcinoma. Cancer Res. 73 (3), 1245–1255. 10.1158/0008-5472.CAN-12-3150 23221387 PMC3563732

[B4] Basavaraju U. Shebl F. M. Palmer A. J. Berry S. Hold G. L. El-Omar E. M. (2015). Cytokine Gene Polymorphisms, Cytokine Levels and the Risk of Colorectal Neoplasia in a Screened Population of Northeast Scotland. Eur. J. Cancer Prev. 24 (4), 296–304. 10.1097/CEJ.0000000000000087 25350634 PMC4411199

[B5] Becker C. Fantini M. C. Wirtz S. Nikolaev A. Lehr H. A. Galle P. R. (2005). IL-6 Signaling Promotes Tumor Growth in Colorectal Cancer. Cell Cycle 4 (2), 217–220. 10.4161/cc.4.2.1413 15655344

[B6] Biao Y. Jiannan H. Yaolan C. Shujie C. Dechun H. Julian Mcclements D. (2020). Identification and Characterization of Antioxidant and Immune-Stimulatory Polysaccharides in Flaxseed hull. Food Chem. 315, 126266. 10.1016/j.foodchem.2020.126266 32000083

[B8] Burrello C. Garavaglia F. Cribiù F. M. Ercoli G. Lopez G. Troisi J. (2018). Therapeutic Faecal Microbiota Transplantation Controls Intestinal Inflammation through IL10 Secretion by Immune Cells. Nat. Commun. 9 (1), 5184. 10.1038/s41467-018-07359-8 30518790 PMC6281577

[B9] Cao J. Tang D. Wang Y. Li X. Hong L. Sun C. (2018). Characteristics and Immune-Enhancing Activity of Pectic Polysaccharides from Sweet Cherry (Prunus Avium). Food Chem. 254, 47–54. 10.1016/j.foodchem.2018.01.145 29548470

[B10] Chattopadhyay I. Dhar R. Pethusamy K. Seethy A. Srivastava T. Sah R. (2021). Exploring the Role of Gut Microbiome in Colon Cancer. Appl. Biochem. Biotechnol. 193 (6), 1780–1799. 10.1007/s12010-021-03498-9 33492552

[B11] Chen R. Li Y. Dong H. Liu Z. Li S. Yang S. (2012). Optimization of Ultrasonic Extraction Process of Polysaccharides from Ornithogalum Caudatum Ait and Evaluation of its Biological Activities. Ultrason. Sonochem. 19 (6), 1160–1168. 10.1016/j.ultsonch.2012.03.008 22525319

[B12] den Besten G. van Eunen K. Groen A. K. Venema K. Reijngoud D. J. Bakker B. M. (2013). The Role of Short-Chain Fatty Acids in the Interplay between Diet, Gut Microbiota, and Host Energy Metabolism. J. Lipid Res. 54 (9), 2325–2340. 10.1194/jlr.R036012 23821742 PMC3735932

[B15] Francescone R. Hou V. Grivennikov S. I. (2015). Cytokines, IBD, and Colitis-Associated Cancer. Inflamm. Bowel Dis. 21 (2), 409–418. 10.1097/MIB.0000000000000236 25563695 PMC4481731

[B16] Fukata M. Chen A. Vamadevan A. S. Cohen J. Breglio K. Krishnareddy S. (2007). Toll-like Receptor-4 Promotes the Development of Colitis-Associated Colorectal Tumors. Gastroenterology 133 (6), 1869–1881. 10.1053/j.gastro.2007.09.008 18054559 PMC2180834

[B17] Giner E. Recio M. C. Ríos J. L. Cerdá-Nicolás J. M. Giner R. M. (2016). Chemopreventive Effect of Oleuropein in Colitis-Associated Colorectal Cancer in C57bl/6 Mice. Mol. Nutr. Food Res. 60 (2), 242–255. 10.1002/mnfr.201500605 26502315

[B18] Grivennikov S. Karin E. Terzic J. Mucida D. Yu G. Y. Vallabhapurapu S. (2009). IL-6 and Stat3 Are Required for Survival of Intestinal Epithelial Cells and Development of Colitis-Associated Cancer. Cancer Cell 15 (2), 103–113. 10.1016/j.ccr.2009.01.001 19185845 PMC2667107

[B19] Hashemifesharaki R. Xanthakis E. Altintas Z. Guo Y. Gharibzahedi S. M. T. (2020). Microwave-assisted Extraction of Polysaccharides from the Marshmallow Roots: Optimization, Purification, Structure, and Bioactivity. Carbohydr. Polym. 240, 116301. 10.1016/j.carbpol.2020.116301 32475574

[B20] Hu D. Bao T. Lu Y. Su H. Ke H. Chen W. (2020). Polysaccharide from Mulberry Fruit (Morus alba L.) Protects against Palmitic-Acid-Induced Hepatocyte Lipotoxicity by Activating the Nrf2/ARE Signaling Pathway. J. Agric. Food Chem. 68 (46), 13016–13024. 10.1021/acs.jafc.9b03335 31537067

[B21] Iguchi T. Kuroda M. Naito R. Watanabe T. Matsuo Y. Yokosuka A. (2017). Structural Characterization of Cholestane Rhamnosides from Ornithogalum Saundersiae Bulbs and Their Cytotoxic Activity against Cultured Tumor Cells. Molecules 22 (8), 1243. 10.3390/molecules22081243 PMC6152286 28757596

[B22] Jackson D. N. Theiss A. L. (2020). Gut Bacteria Signaling to Mitochondria in Intestinal Inflammation and Cancer. Gut Microbes 11 (3), 285–304. 10.1080/19490976.2019.1592421 30913966 PMC7524274

[B23] Ji X. Peng Q. Wang M. (2018). Anti-colon-cancer Effects of Polysaccharides: A Mini-Review of the Mechanisms. Int. J. Biol. Macromol 114, 1127–1133. 10.1016/j.ijbiomac.2018.03.186 29627471

[B24] Kang M. Martin A. (2017). Microbiome and Colorectal Cancer: Unraveling Host-Microbiota Interactions in Colitis-Associated Colorectal Cancer Development. Semin. Immunol. 32, 3–13. 10.1016/j.smim.2017.04.003 28465070

[B25] Kim S. Keku T. O. Martin C. Galanko J. Woosley J. T. Schroeder J. C. (2008). Circulating Levels of Inflammatory Cytokines and Risk of Colorectal Adenomas. Cancer Res. 68 (1), 323–328. 10.1158/0008-5472.CAN-07-2924 18172326 PMC2675825

[B26] Klampfer L. (2008). The Role of Signal Transducers and Activators of Transcription in colon Cancer. Front. Biosci. 13, 2888–2899. 10.2741/2893 17981761

[B27] Knüpfer H. Preiss R. (2010). Serum Interleukin-6 Levels in Colorectal Cancer Patients-Aa Summary of Published Results. Int. J. Colorectal Dis. 25 (2), 135–140. 10.1007/s00384-009-0818-8 19898853

[B28] Li M. Y. Luo H. J. Wu X. Liu Y. H. Gan Y. X. Xu N. (2019). Anti-Inflammatory Effects of Huangqin Decoction on Dextran Sulfate Sodium-Induced Ulcerative Colitis in Mice through Regulation of the Gut Microbiota and Suppression of the Ras-PI3K-Akt-HIF-1α and NF-κB Pathways. Front. Pharmacol. 10, 1552. 10.3389/fphar.2019.01552 32038240 PMC6984456

[B29] Li Q. Wang W. Zhu Y. Chen Y. Zhang W. Yu P. (2017). Structural Elucidation and Antioxidant Activity a Novel Se-Polysaccharide from Se-Enriched Grifola Frondosa. Carbohydr. Polym. 161, 42–52. 10.1016/j.carbpol.2016.12.041 28189245

[B30] Li R. Chen Y. Shi M. Xu X. Zhao Y. Wu X. (2016). Gegen Qinlian Decoction Alleviates Experimental Colitis via Suppressing TLR4/NF-κB Signaling and Enhancing Antioxidant Effect. Phytomedicine 23 (10), 1012–1020. 10.1016/j.phymed.2016.06.010 27444346

[B31] Liu F. Liu A. Lu X. Zhang Z. Xue Y. Xu J. (2019). Dysbiosis Signatures of the Microbial Profile in Tissue from Bladder Cancer. Cancer Med. 8 (16), 6904–6914. 10.1002/cam4.2419 31568654 PMC6854010

[B32] Liu G. Liang L. Yu G. Li Q. (2018a). Pumpkin Polysaccharide Modifies the Gut Microbiota during Alleviation of Type 2 Diabetes in Rats. Int. J. Biol. Macromol 115, 711–717. 10.1016/j.ijbiomac.2018.04.127 29702167

[B33] Liu J. Hao W. He Z. Kwek E. Zhu H. Ma N. (2021a). Blueberry and cranberry Anthocyanin Extracts Reduce Bodyweight and Modulate Gut Microbiota in C57BL/6 J Mice Fed with a High-Fat Diet. Eur. J. Nutr. 60 (5), 2735–2746. 10.1007/s00394-020-02446-3 33392758

[B80] Liu J. Wu S. Chen Y. Li Q. Su L. Yang Y. (2021b). Sargassum fusiforme Alginate Relieves Hyperglycemia and Modulates Intestinal Microbiota and Metabolites in Type 2 Diabetic Mice. Nutrients 13 (8). 10.3390/nu13082887 PMC8398017 34445047

[B79] Liu J. Wu S. Y. Chen L. Li Q. J. Shen Z. Y. Jin L. (2020). Different Extraction Methods Bring About Distinct Physicochemical Properties And Antioxidant Activities Of Sargassum Fusiforme Fucoidans. Int. J. Biol. Macromol 155, 1385–1392. 10.1016/j.ijbiomac.2019.11.113 31733246

[B34] Liu L. Q. Li H. S. Nie S. P. Shen M. Y. Hu J. L. Xie M. Y. (2018b). Tea Polysaccharide Prevents Colitis-Associated Carcinogenesis in Mice by Inhibiting the Proliferation and Invasion of Tumor Cells. Int. J. Mol. Sci. 19 (2), 506. 10.3390/ijms19020506 PMC5855728 29419740

[B35] Liu L. Q. Nie S. P. Shen M. Y. Hu J. L. Yu Q. Gong D. (2018c). Tea Polysaccharides Inhibit Colitis-Associated Colorectal Cancer via Interleukin-6/STAT3 Pathway. J. Agric. Food Chem. 66 (17), 4384–4393. 10.1021/acs.jafc.8b00710 29656647

[B81] Lou M. Cao A. Jin C. Mi K. Xiong X. Zeng Z. (2021). Deviated And Early Unsustainable Stunted Development Of Gut Microbiota In Children With Autism Spectrum Disorder. Gut. 10.1136/gutjnl-2021-325115 PMC9279844 34930815

[B36] Luo L. Sun W. Zhu W. Li S. Zhang W. Xu X. (2021). BCAT1 Decreases the Sensitivity of Cancer Cells to Cisplatin by Regulating mTOR-Mediated Autophagy via Branched-Chain Amino Acid Metabolism. Cell Death Dis 12 (2), 169. 10.1038/s41419-021-03456-7 33568627 PMC7876012

[B37] Ma F. Wang R. Li X. Kang W. Bell A. E. Zhao D. (2020). Physical Properties of Mucilage Polysaccharides from Dioscorea Opposita Thunb. Food Chem. 311, 126039. 10.1016/j.foodchem.2019.126039 31869644

[B38] Marongiu L. Landry J. J. M. Rausch T. Abba M. L. Delecluse S. Delecluse H. J. (2021). Metagenomic Analysis of Primary Colorectal Carcinomas and Their Metastases Identifies Potential Microbial Risk Factors. Mol. Oncol. 15, 3363–3384. 10.1002/1878-0261.13070 34328665 PMC8637581

[B39] Matsunaga Y. Hasei S. Yamamotoya T. Honda H. Kushiyama A. Sakoda H. (2021). Pathological Role of Pin1 in the Development of DSS-Induced Colitis. Cells 10 (5), 1230. 10.3390/cells10051230 34067858 PMC8155908

[B40] Meng Q. Li Y. Xiao T. Zhang L. Xu D. (2017). Antioxidant and Antibacterial Activities of Polysaccharides Isolated and Purified from Diaphragma Juglandis Fructus. Int. J. Biol. Macromol 105 (Pt 1), 431–437. 10.1016/j.ijbiomac.2017.07.062 28711614

[B41] Papapietro O. Teatero S. Thanabalasuriar A. Yuki K. E. Diez E. Zhu L. (2013). R-spondin 2 Signalling Mediates Susceptibility to Fatal Infectious Diarrhoea. Nat. Commun. 4, 1898. 10.1038/ncomms2816 23695692 PMC4844535

[B43] Ren Y. Geng Y. Du Y. Li W. Lu Z. M. Xu H. Y. (2018). Polysaccharide of Hericium erinaceus Attenuates Colitis in C57BL/6 Mice via Regulation of Oxidative Stress, Inflammation-Related Signaling Pathways and Modulating the Composition of the Gut Microbiota. J. Nutr. Biochem. 57, 67–76. 10.1016/j.jnutbio.2018.03.005 29677563

[B44] Robles A. I. Traverso G. Zhang M. Roberts N. J. Khan M. A. Joseph C. (2016). Whole-Exome Sequencing Analyses of Inflammatory Bowel Disease-Associated Colorectal Cancers. Gastroenterology 150 (4), 931–943. 10.1053/j.gastro.2015.12.036 26764183 PMC5270616

[B45] Sanders K. Moran Z. Shi Z. Paul R. Greenlee H. (2016). Natural Products for Cancer Prevention: Clinical Update 2016. Semin. Oncol. Nurs. 32 (3), 215–240. 10.1016/j.soncn.2016.06.001 27539278

[B46] Siegel R. L. Miller K. D. Fuchs H. E. Jemal A. (2021). Cancer Statistics, 2021. CA A. Cancer J. Clin. 71 (1), 7–33. 10.3322/caac.21654 33433946

[B47] Song H. Wang W. Shen B. Jia H. Hou Z. Chen P. (2018). Pretreatment with Probiotic Bifico Ameliorates Colitis-Associated Cancer in Mice: Transcriptome and Gut flora Profiling. Cancer Sci. 109 (3), 666–677. 10.1111/cas.13497 29288512 PMC5834773

[B48] Song M. Mehta R. S. Wu K. Fuchs C. S. Ogino S. Giovannucci E. L. (2016). Plasma Inflammatory Markers and Risk of Advanced Colorectal Adenoma in Women. Cancer Prev. Res. (Phila) 9 (1), 27–34. 10.1158/1940-6207.CAPR-15-0307 26511487 PMC4706809

[B49] Su Y. Li L. (2020). Structural Characterization and Antioxidant Activity of Polysaccharide from Four Auriculariales. Carbohydr. Polym. 229, 115407. 10.1016/j.carbpol.2019.115407 31826485

[B50] Sun Y. O'Riordan M. X. (2013). Regulation of Bacterial Pathogenesis by Intestinal Short-Chain Fatty Acids. Adv. Appl. Microbiol. 85, 93–118. 10.1016/B978-0-12-407672-3.00003-4 23942149 PMC4029053

[B51] Sun Y. D. Wang Z. H. Ye Q. S. (2013). Composition Analysis and Anti-proliferation Activity of Polysaccharides from Dendrobium Chrysotoxum. Int. J. Biol. Macromol 62, 291–295. 10.1016/j.ijbiomac.2013.08.046 24012699

[B52] Tan J. McKenzie C. Potamitis M. Thorburn A. N. Mackay C. R. Macia L. (2014). The Role of Short-Chain Fatty Acids in Health and Disease. Adv. Immunol. 121, 91–119. 10.1016/B978-0-12-800100-4.00003-9 24388214

[B53] Ullman T. A. Itzkowitz S. H. (2011). Intestinal Inflammation and Cancer. Gastroenterology 140 (6), 1807–1816. 10.1053/j.gastro.2011.01.057 21530747

[B54] Uronis J. M. Mühlbauer M. Herfarth H. H. Rubinas T. C. Jones G. S. Jobin C. (2009). Modulation of the Intestinal Microbiota Alters Colitis-Associated Colorectal Cancer Susceptibility. PLoS One 4 (6), e6026. 10.1371/journal.pone.0006026 19551144 PMC2696084

[B55] van der Beek C. M. Dejong C. H. C. Troost F. J. Masclee A. A. M. Lenaerts K. (2017). Role of Short-Chain Fatty Acids in Colonic Inflammation, Carcinogenesis, and Mucosal protection and Healing. Nutr. Rev. 75 (4), 286–305. 10.1093/nutrit/nuw067 28402523

[B56] van der Lugt B. van Beek A. A. Aalvink S. Meijer B. Sovran B. Vermeij W. P. (2019). Akkermansia Muciniphila Ameliorates the Age-Related Decline in Colonic Mucus Thickness and Attenuates Immune Activation in Accelerated Aging Ercc1 -/Δ7 Mice. Immun. Ageing 16, 6. 10.1186/s12979-019-0145-z 30899315 PMC6408808

[B57] Waldner M. J. Neurath M. F. (2015). Mechanisms of Immune Signaling in Colitis-Associated Cancer. Cell Mol Gastroenterol Hepatol 1 (1), 6–16. 10.1016/j.jcmgh.2014.11.006 28247866 PMC5301162

[B58] Wang R. Chen P. Jia F. Tang J. Ma F. (2012). Optimization of Polysaccharides from Panax Japonicus C.A. Meyer by RSM and its Anti-oxidant Activity. Int. J. Biol. Macromol 50 (2), 331–336. 10.1016/j.ijbiomac.2011.12.023 22214823

[B59] Wu P. Zhang G. Zhao J. Chen J. Chen Y. Huang W. (2018). Profiling the Urinary Microbiota in Male Patients with Bladder Cancer in China. Front Cel Infect Microbiol 8, 167. 10.3389/fcimb.2018.00167 PMC5990618 29904624

[B60] Wu S. Zhang X. Liu J. Song J. Yu P. Chen P. (2019). Physicochemical Characterization of Sargassum Fusiforme Fucoidan Fractions and Their Antagonistic Effect against P-Selectin-Mediated Cell Adhesion. Int. J. Biol. Macromol 133, 656–662. 10.1016/j.ijbiomac.2019.03.218 30930270

[B61] Xia W. Khan I. Li X. A. Huang G. Yu Z. Leong W. K. (2020a). Adaptogenic Flower Buds Exert Cancer Preventive Effects by Enhancing the SCFA-Producers, Strengthening the Epithelial Tight junction Complex and Immune Responses. Pharmacol. Res. 159, 104809. 10.1016/j.phrs.2020.104809 32502642

[B62] Xia X. Wu W. K. K. Wong S. H. Liu D. Kwong T. N. Y. Nakatsu G. (2020b). Bacteria Pathogens Drive Host Colonic Epithelial Cell Promoter Hypermethylation of Tumor Suppressor Genes in Colorectal Cancer. Microbiome 8 (1), 108. 10.1186/s40168-020-00847-4 32678024 PMC7367367

[B63] Yu H. Lee H. Herrmann A. Buettner R. Jove R. (2014). Revisiting STAT3 Signalling in Cancer: New and Unexpected Biological Functions. Nat. Rev. Cancer 14 (11), 736–746. 10.1038/nrc3818 25342631

[B64] Yu H. Pardoll D. Jove R. (2009). STATs in Cancer Inflammation and Immunity: a Leading Role for STAT3. Nat. Rev. Cancer 9 (11), 798–809. 10.1038/nrc2734 19851315 PMC4856025

[B65] Yu Y. Lu J. Sun L. Lyu X. Chang X. Y. Mi X. (2021). Akkermansia Muciniphila: A Potential Novel Mechanism of Nuciferine to Improve Hyperlipidemia. Biomed. Pharmacother. 133, 111014. 10.1016/j.biopha.2020.111014 33246225

[B66] Yuan X. Xue J. Tan Y. Yang Q. Qin Z. Bao X. (2021). Albuca Bracteate Polysaccharides Synergistically Enhance the Anti-tumor Efficacy of 5-Fluorouracil against Colorectal Cancer by Modulating β-Catenin Signaling and Intestinal Flora. Front. Pharmacol. 12, 736627. 10.3389/fphar.2021.736627 34552494 PMC8450769

[B67] Zeng F. Chen W. He P. Zhan Q. Wang Q. Wu H. (2020). Structural Characterization of Polysaccharides with Potential Antioxidant and Immunomodulatory Activities from Chinese Water Chestnut Peels. Carbohydr. Polym. 246, 116551. 10.1016/j.carbpol.2020.116551 32747236

[B68] Zhang J. Zhou H. C. He S. B. Zhang X. F. Ling Y. H. Li X. Y. (2021). The Immunoenhancement Effects of Sea Buckthorn Pulp Oil in Cyclophosphamide-Induced Immunosuppressed Mice. Food Funct. 12 (17), 7954–7963. 10.1039/d1fo01257f 34251375

[B69] Zhang Y. Fang F. Fan K. Zhang Y. Zhang J. Guo H. (2017). Effective Cytotoxic Activity of OSW-1 on colon Cancer by Inducing Apoptosis *In Vitro* and *In Vivo* . Oncol. Rep. 37 (6), 3509–3519. 10.3892/or.2017.5582 28440433

[B70] Zhang Y. Liu Q. Yu Y. Wang M. Wen C. He Z. (2020). Early and Short-Term Interventions in the Gut Microbiota Affects Lupus Severity, Progression, and Treatment in MRL/lpr Mice. Front. Microbiol. 11, 628. 10.3389/fmicb.2020.00628 32346376 PMC7171286

[B71] Zhao H. M. Wang Y. Huang X. Y. Huang M. F. Xu R. Yue H. Y. (2016). Astragalus Polysaccharide Attenuates Rat Experimental Colitis by Inducing Regulatory T Cells in Intestinal Peyer's Patches. World J. Gastroenterol. 22 (11), 3175–3185. 10.3748/wjg.v22.i11.3175 27003994 PMC4789992

[B72] Zhong L. Zhang X. Covasa M. (2014). Emerging Roles of Lactic Acid Bacteria in protection against Colorectal Cancer. World J. Gastroenterol. 20 (24), 7878–7886. 10.3748/wjg.v20.i24.7878 24976724 PMC4069315

[B73] Zhou Y. Garcia-Prieto C. Carney D. A. Xu R. H. Pelicano H. Kang Y. (2005). OSW-1: a Natural Compound with Potent Anticancer Activity and a Novel Mechanism of Action. J. Natl. Cancer Inst. 97 (23), 1781–1785. 10.1093/jnci/dji404 16333034

[B74] Zhu H. Li Y. R. (2012). Oxidative Stress and Redox Signaling Mechanisms of Inflammatory Bowel Disease: Updated Experimental and Clinical Evidence. Exp. Biol. Med. (Maywood) 237 (5), 474–480. 10.1258/ebm.2011.011358 22442342

[B75] Zhu H. C. Jia X. K. Fan Y. Xu S. H. Li X. Y. Huang M. Q. (2021). Alisol B 23-Acetate Ameliorates Azoxymethane/Dextran Sodium Sulfate-Induced Male Murine Colitis-Associated Colorectal Cancer via Modulating the Composition of Gut Microbiota and Improving Intestinal Barrier. Front. Cel Infect Microbiol 11, 640225. 10.3389/fcimb.2021.640225 PMC8117151 33996624

[B76] Zorron Cheng Tao Pu L. Yamamoto K. Honda T. Nakamura M. Yamamura T. Hattori S. (2020). Microbiota Profile Is Different for Early and Invasive Colorectal Cancer and Is Consistent throughout the colon. J. Gastroenterol. Hepatol. 35 (3), 433–437. 10.1111/jgh.14868 31609493

[B77] Zou Q. Zhang X. Liu X. Li Y. Tan Q. Dan Q. (2020). Ficus Carica Polysaccharide Attenuates DSS-Induced Ulcerative Colitis in C57BL/6 Mice. Food Funct. 11 (7), 6666–6679. 10.1039/d0fo01162b 32658237

[B78] Zou Y. Chen T. (2020). Engineered Akkermansia Muciniphila: A Promising Agent against Diseases (Review). Exp. Ther. Med. 20 (6), 285. 10.3892/etm.2020.9415 33209129 PMC7668130

