# Supplementary material for: Albuca Bracteata Polysaccharides Attenuate AOM/DSS Induced Colon Tumorigenesis via Regulating Oxidative Stress, Inflammation and Gut Microbiota in Mice
Source: Front Pharmacol. 2022 Feb 21;13:833077. doi: 10.3389/fphar.2022.833077 (PMC8899018; doi:10.3389/fphar.2022.833077)
Supplement: Supplementary file 1 [file DataSheet1.docx]

*Supplementary Information*

Table S1. Physicochemical Properties of ABP

Table S2. The evaluation details of the histological score

Figure S1. Effects of ABP alone on mice

Figure S2. Alpha and Beta diversity index in each group (treatment with ABP for 3 weeks)

Figure S3. The relative abundance of representative gut microbiota in Phylum, Family and Genus level (treatment with ABP for 3 weeks)

Figure S4. LEfSe analysis of bacterial taxa differences in each group (treatment with ABP for 3 weeks)

Figure S5. Differences in representative gut microbiota between 3 and l1 weeks treatment

Table S1. Physicochemical Properties of ABP

| Molecular weight (kDa) | Total carbohydrate (%) | Uronic acid (%) | Protein (%) | | Molar ratios monosaccharide (mol%) | | | | | |
| --- | --- | --- | --- | --- | --- | --- | --- | --- | --- | --- |
|  |  |  |  |  | Glc | Man | Gal | Xyl | GalA | GlcA |
| 18.3 | 92.58 | 1.63 | | 1.70 | 37.8 | 8 | 2.5 | 1.7 | 1 | 1 |

Table S2. The evaluation details of the histological score.

| Histological score | | |
| --- | --- | --- |
| Mucosal epithelium | 0 | No mucosa inflammation |
|  | 1 | Loss of <5% of the epithelial surface |
|  | 2 | Loss of 5-10% of the epithelial surface |
|  | 3 | Loss of >10% of the epithelial surface |
| Integrity of crypts | 0 | Intact crypts |
|  | 1 | Loss of <10% crypts |
|  | 2 | Loss of 10-20% crypts |
|  | 3 | Loss of >20% of crypts |
| Cell infiltrate and edema | 0 | None |
|  | 1 | Mild |
|  | 2 | moderate |
|  | 3 | severe |
| Goblet cells depletion | 0 | Absent |
|  | 1 | Present |

Figure S1. Effects of ABP alone on mice. (A-B) Colon lengths of mice in each group. (C) Body weights of mice in each group. (D) Micrographs of HE-stained colon tissues.

Figure S2. Alpha and Beta diversity index in each group (treatment with ABP for 3 weeks). (A-B) Analysis of alpha diversity; (A) Simpson index. (B) Shannon index. (C-D) Analysis of beta diversity. principal coordination analysis (PCoA).

Figure S3. The relative abundance of representative gut microbiota in Phylum, Family and Genus level (treatment with ABP for 3 weeks). (A-C) Heatmap of gut microbiota based at the phylum, family and genus level.

Figure S4. LEfSe analysis of bacterial taxa differences in each group (treatment with ABP for 3 weeks). (A-B) LEfSe analysis of bacterial taxa differences in each group. (C) Box plots demonstrating the characteristic bacteria at genus levels.

Figure S5. Differences in representative gut microbiota between 3 and l1 weeks treatment.
